# Ethnomedicine study on traditional medicinal plants in the Wuliang Mountains of Jingdong, Yunnan, China

**DOI:** 10.1186/s13002-019-0316-1

**Published:** 2019-08-19

**Authors:** Lunlun Gao, Neng Wei, Guoping Yang, Zhenxian Zhang, Guizhou Liu, Chuantao Cai

**Affiliations:** 1 Key Laboratory of Tropical Plant Resources and Sustainable Use, Xishuangbanna Tropical Botanical Garden, Chinese Academy of Sciences, Mengla, 666303 Yunnan China; 2 Huazhong Agricultural University, Wuhan, 430070 Hubei China; 3 University of the Chinese Academy of Science, Beijing, 100049 China; 4 Wuhan Botanical Garden, Chinese Academy of Sciences, Wuhan, 430074 Hubei China

**Keywords:** Ethnomedicine, Traditional knowledge, Medicinal plants, Wuliang Mountains region

## Abstract

**Background:**

The Wuliang Mountains of the Jingdong region is a settlement area of the Yi community located in south-western Yunnan Province in China. Due to its unique geographical location, this area harbours abundant medicinal plant resources. The medicinal plants used by the local people have a long history and play an important role in their daily life. During the long-term mixed lifestyle, the knowledge of traditional medicinal plants in different communities has been assimilated to some extent. Therefore, this paper is based on ethnobotanical investigations to document traditional medicinal plants used by local people and discuss the differences between the Yi and Han communities in the study area.

**Methods:**

Data on traditional medicinal plants were collected from September 2016 to August 2017 in the Yi autonomous county of Jingdong. Seven townships and 16 villages were selected for the field investigations. Information was obtained through key informant interviews. A total of 44 key informants were interviewed, and all of them were herbalists or herbal sellers.

**Results:**

In this study, a total of 302 traditional medicinal plant species belonging to 117 families and 252 genera were investigated and documented, most of which were obtained from herbalists. Although family Asteraceae was the most prevalent, with 27 species, the most commonly utilized species were members of family Papaveraceae, *Dactylicapnos scandens* (D. Don) Hutch*.*, which is used as an antipyretic drug. Herbs comprised half of the total number of species, and the whole plant is the most frequently utilized plant part. The plants were used to treat more than 93 human diseases, with antipyretic drugs being the most common form of herbal medicine. The traditional medicinal plants used in the study area possess a high ratio of being documented in the literature. According to the analysis, the *Chinese Pharmacopoeia* recorded 76 species and the *Resources of Traditional Chinese Medicine* recorded 233 species of traditional medicinal plants. By evaluating the endangered status of the traditional medicinal plants in the study area, we found good conservation status of the cited medicinal plants. Regarding the similarity between the communities, there were significant differences between the Yi and Han communities, as indicated by the Jaccard similarity index (0.232).

**Conclusions:**

Medicinal plants are the embodiment of wisdom from our ancestors and play a significant role in treating various human disorders. As one of the birthplaces of Yi medicine, the study area possesses a high species diversity of traditional medicinal plants used by local people. With the rapid development of modern medicine, however, the inheritance of this valuable culture is facing enormous threats even though its potential value has not yet been fully explored. Therefore, some effective protection measures should be taken, and some modern techniques should be implemented to prove the safety and improve the scientific acceptance of the traditional medicinal plants.

**Electronic supplementary material:**

The online version of this article (10.1186/s13002-019-0316-1) contains supplementary material, which is available to authorized users.

## Introduction

According to the World Health Organization (WHO), approximately 65–80% of the world’s population in developing countries essentially depends on plants for their primary health care [1]. China has kept the tradition of using herbs to treat diseases since ancient times, and this was the principal method for the treatment of disease before the popularization of modern medicine. For the remote minority, in particular, traditional medicinal plants hold a significant position in their daily livelihood. The value hidden behind them deserves to be explored. However, the sustainable utilization of traditional medicinal plants is threatened by the rapid development of the social economy in China. Although knowledge regarding traditional medicinal plants has been documented in some regions [2–4], more research is needed to document the knowledge about traditional medicinal plant usages, and urgent conservation measures should be implemented as well [5].

The Yi community is one of the oldest communities in China and lives in the Hengduan region, which has been rich in medicinal plants for a long time. This community created a unique traditional system of medicine with its own theory as it struggled with diseases. Because of the blockage of the traditional knowledge inheritance within the Yi community, such knowledge has only spread within the same clade, family or region, resulting in unbalanced development in different areas [6]. Compared with the adjacent Chuxiong and Shuangbai districts, which have both been systematically studied [7], however, the traditional medicinal plants of the Yi community in Jingdong are still under-researched.

In contrast to other clades, the Yi community in the Wuliang Mountains have no particular wordage. For this reason, the study of the traditional medicinal plants in this region is necessary and urgent [8]. In this survey, the ethnomedicine approach of the key informant interview is used to assess the utilization of traditional medicinal plants by local people.

## Study area and data collection

### Study area

**Fig. 1:**
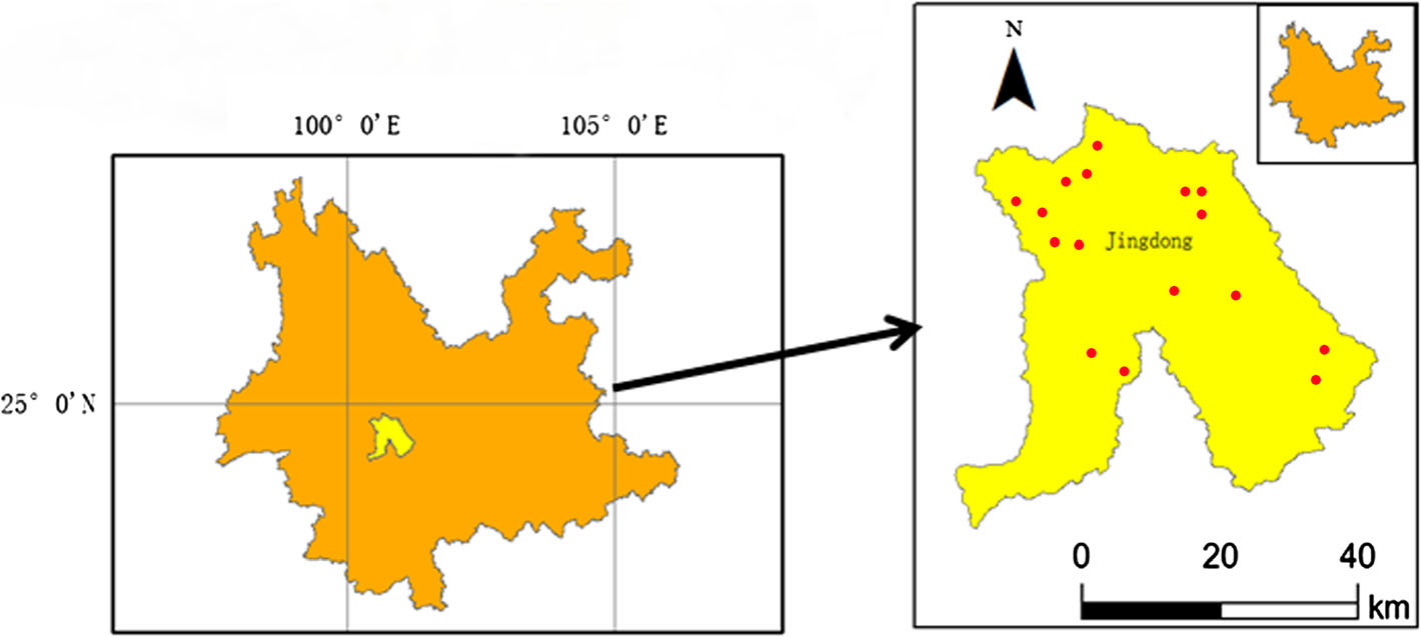
Map of the study area showing the location of villages

The Wuliang Mountains are situated in the southwest of Yunnan Province and are located at 23°57′–24°44′ N latitude and 100°22′–101°04′ E longitude (Fig. 1). As an extension of the Hengduan mountain range, the Wuliang Mountains stretch for 89 km from north to south, with an average altitude above 2000 m. The northwestern side of Wuliang Mountains lies in the alternating transition zone between the eastern Asiatic and Paleotropical flora regions, and the southeastern part lies in the alternating transition zone between the China-Japan plant subregion and the China-Himalayan plant subregion. The Wuliang Mountains belong to the western monsoon climate zone, which is characterized by a distinctive south Asian monsoon with obvious wet and dry seasons, harbour plants that exhibit continuous blooming and have the climatic characteristics of plateaus at low latitudes [9]. These unique geographical and climatic conditions result in rich plant diversity in this area. As mentioned by Peng [10], there are more than 300 types of medicinal plants with significant research value.

The Yi autonomous county of Jingdong has a total population of 35.55 million. The Han ethnic group comprises 18.35 million (50.21%), while the Yi comprises 15.46 million (42.36%) of the total population [11]. The Yi community in Jingdong is distributed on both sides of the Wuliang Mountains. As a clade of the Yi ethnic group, the Yi autonomous county of Jingdong is one of the settlements and birthplaces of Yi community medicine [10], with a lifestyle of mixed habitation for a long time. The mutual effects of the two ethnicities have resulted in the fusion of culture and utilization of medicinal plants.

### Data collection

Ethnobotanical data were collected from September 2016 to August 2017 in the Yi autonomous county of Jingdong, southwest Yunnan. Seven townships and 16 villages distributed on the two sides of the Wuliang Mountains were selected for the field investigations (Additional file 1). Information was collected via key informant interviews. A total of 44 informants were interviewed in the study area, with all the informants being local inhabitants with a profession of herbalist or seller of herbs and who embrace lots of medicinal knowledge. Their gender, age, nationality, education level and occupation were recorded. Ethnobotanical investigations were carried out to collect data on the medicinal plants used to treat human ailments, including their Latin name, Chinese name, local name, family name, life form, plant parts used, preparation method and medicinal effect. All plants were identified according to the *Flora Reipublicae Popularis Sinicae* [12]. Voucher specimens of the plants cited by informants were collected and deposited at the Herbarium of Xishuangbanna Tropical Botanical Garden (HITBC).

## Results and discussion

### Profile of informants

The constituent information regarding age, gender, nationality, education and occupation of informants is shown in Table 1. Most of the informants were males, and they played a significant role in the activities of collecting and using traditional medicinal plants. Females only had some knowledge about postpartum diseases.

**Table 1:** The background information of informants in this study

Gender	
Male	36 (81.82%)
Female	8 (18.18%)
Age	
20~29	3 (6.82%)
30~39	2 (4.55%)
40~49	19 (43.18%)
50~59	8 (18.18%)
60~69	9 (20.45%)
70~79	3 (6.82%)
Nation	
Yi nationality	31 (70.45%)
Han nationality	13 (29.55%)
Education level	
Primary school	20 (45.45%)
Secondary school	20 (45.45%)
College/university	4 (9.09%)
Profession	
Herbalist	12 (27.27%)
Non-herbalist	32 (72.73%)

All of the informants were split into six age groups, with an average of 49.64 years old. The 40–49-year-old group comprised 43.18%. The Yi population accounted for 70.45%. The educational level of the informants centred on primary and middle school. In our study, 5 out of 12 herbalists who participated in the interview changed their profession, and the 7 herbalists left were still engaged in this profession. In addition, only 2 herbalists had successors, since no young people were willing to engage in this hard and difficult work. According to our investigation, the main reason for this observation is the fact that the low income as an herbalist makes it difficult to make a living. The trend of this phenomenon poses a significant threat to the inheritance of this traditional culture.

### Traditional medicinal plant diversity in the study

This study recorded 302 medicinal plant species belonging to 252 genera and 117 families that were used to treat more than 93 ailments (Table 2). The traditional medicinal plants showed high diversity in terms of the composition of species at the family and genus level, with the single-species family and the single-species genus having an absolute advantage in number. Among these medicinal plants, the most species-rich family was Asteraceae, represented by 27 species, followed by family Fabaceae, with 14 species, which is similar to Li [3]. The main reason for this result is likely the abundance of species in these two families. Furthermore, the richest plant genera were *Cinnamomum*, *Aconitum*, *Artemisia* and *Polygonum*, each represented by 4 species. The most commonly utilized species is *Dactylicapnos scandens* (D. Don) Hutch*.*, which belongs to Papaveraceae and is used as an antipyretic drug.

**Table 2:** The inventory of medicinal plants traditionally used by local people

Vernacular name	Family name	Latin name	Habit	Part used	Medicinal use	Voucher number
Dakonghua	Malvaceae	*Abelmoschus manihot* var. *pungens* (Roxb.) Hochr.	Shrub	Root	Unknown swollen	GLL0162
Sheyao	Compositae	*Achillea millefolium* Linn.	Shrub	Root, leaf, whole plant	Snake venom, common cold, meningitis	GLL00113
Tuniuxi	Amaranthaceae	*Achyranthes aspera* Linn.	Herb	Whole plant	Bone-setting	GLL0262
Tongchuicao	Compositae	*Acmella calva* (DC.) R.K.Jansen	Herb	Whole plant	Traumatic injury	GLL00123
Jinwu	Ranunculaceae	*Aconitum austroyunnanen*se W.T. Wang	Herb	Whole plant, Root	Cold drugs, traumatic injury	GLL0127
Xueshangyizhihao	Ranunculaceae	*Aconitum brachypodum* Diels	Herb	Root	Traumatic injury, rheumatism	GLL0129
Caowu	Ranunculaceae	*Aconitum carmichaelii* Debx.	Herb	Root	Bone-setting, traumatic injury, digestive, general aching, common cold, hyperosteogeny, rheumatism	GLL0126
Daduwu	Ranunculaceae	*Aconitum scaposum Franch*.var.*hupehanum* Rapaics	Herb	Root	Traumatic injury	GLL0128
Changpu	Acoraceae	*Acorus calamus*	Herb	Root	Digestive	GLL055
Shichangpu	Araceae	*Acorus gramineus* Sol. ex Aiton	Herb	Whole plant	Digestive	GLL0082
Zhuzongcao	Adiantaceae	*Adiantum bonatianum* Brause	Herb	Whole plant	Cystitis, diuretic	GLL098
Suoluo	Hippocastanaceae	*Aesculus chinensis* Bunge	Tree	Root, stem	Gastroenteritis	GLL082
Honghualuobo	Ericaceae	*Agapetes hosseana* Diels	Shrub	Root	Traumatic injury	GLL0184
Shujidan	Ericaceae	*Agapetes mannii* Hemsl.	Shrub	Root	Traumatic injury, rheumatism palpitation	GLL0182
Huoxiang	Labiatae	*Agastache rugosa* (Fisch. et Mey.) O. Kuntze	Herb	Whole plant	Relieving cough, pneumonia, ventilation, common cold, digestive	GLL0041
Jima	Agavaceae	*Agave sisalana* Perrine ex Engelm.	Herb	Root	Common cold (for child)	GLL075
Daheicao	Compositae	*Ageratina adenophora* (Spreng.) R. M. King et H. Rob.	Herb	Whole plant	Common cold, gastroenteritis	GLL00119
Xianhecao	Rosaceae	*Agrimonia pilosa* var. *nepalensis* (D. Don) Nakai	Herb	Whole plant, root	Haemostasis, flooding, gastroenteritis, dysentery	GLL0021
Yexiahua	Compositae	*Ainsliaea pertyoides* Franch.	Herb	Whole plant	Traumatic injury, gynecologic diseases	GLL00116
Shenyancao	Compositae	*Ainsliaea spicata* Vaniot	Herb	Root	Heat-clearing and detoxifying, nephritis	GLL00115
Mutong	Lardizabalaceae	*Akebia quinata* (Houtt.) Decne.	Woody climber	Stem, leaf	Hyperlipidemia, hypertension	GLL079
Handonggua	Betulaceae	Alnus nepalensis	Tree	Bark, leaf	Gastroenteritis	GLL067
Dayedengtai	Apocynaceae	*Alstonia scholaris* (Linn.) R. Br.	Tree	Leaf	Relieving cough, trachitis	GLL0152
Moyu	Araceae	*Amorphophallus konjac* K. Koch	Herb	Root	Digestive, obesity	GLL0088
Yeputao	Vitaceae	*Ampelopsis glandulosa* (Wall.) Momiy.	Woody climber	Whole plant	Blood phobia	GLL0242
Taoren	Rosaceae	*Amygdalus davidiana* (Carrière) de Vos ex Henry	Tree	Nutlet, bark, leaf	Traumatic injury, rheumatism gastroenteritis, toothache	GLL0028
Huzhangcao	Ranunculaceae	*Anemone rivularis* Buch.-Ham.	Herb	Root	Hepatitis, gastroenteritis	GLL01210
Danggui	Umbelliferae	*Angelica sinensis* (Oliv.) Diels	Herb	Root	Tonic, traumatic injury	GLL0117
Sanfensan	Solanaceae	*Anisodus acutangulus* C. Y. Wu et C. Chen ex C. Chen et C. L. Chen	Herb	Leaf, whole plant, root	Bone-setting, traumatic injury antiphlogosis, rheumatism	GLL0204
Baiyundougen	Fabaceae	*Apios carnea* (Wall.) Benth. ex Baker	Woody climber	Root	Digestive	GLL0037
Niubang	Compositae	*Arctium lappa* L.	Herb	Root	Nephritis	GLL00110
Baoziyanjinghua	Myrsinaceae	*Ardisia crenata* Sims	Shrub	Root, whole plant	Common cold antiphlogosis gastroenteritis	GLL0323
Zijinniu	Myrsinaceae	*Ardisia japonica* (Thunb.) Bl.	Shrub	Root	Heat-clearing and detoxifying	GLL0322
Binlang	Palmae	*Areca catechu* L.	Tree	Fruit	Digestive	GLL0431
Dahanyao	Aristolochiaceae	*Aristolochia cucurbitoides* C.F. Liang	Herbaceous liane	Root	Digestive	GLL0442
Tumuxiang	Aristolochiaceae	*Aristolochia debilis* Sieb. et Zucc.	Herbaceous liane	Root	Gastroenteritis. Relieving cough, gastroenteritis	GLL0441
Qingmuxiang	Aristolochiaceae	*Aristolochia transsecta* (Chatterjee) C. Y. Wu ex S. M. Hwang	Woody climber	Root	Gastroenteritis	GLL0443
Aihao	Compositae	Artemisia argyi	Herb	Whole plant	Gynecologic diseases	GLL00126
Yinchenhao	Compositae	*Artemisia capillaris* Thunb.	Herb	Whole plant	Cholecystitis	GLL0016
Haozi	Compositae	*Artemisia carvifolia* Buch.-Ham. ex Roxb.	Herb	Whole plant, root, leaf	Common cold, antiphlogosis, haemostasis, heat-clearing and detoxifying Gynecologic diseases, gastroenteritis, haemostasis	GLL0014
Pingtouhao	Compositae	*Artemisia japonica* Thunb.	Herb	Whole plant, root, leaf	Common cold, hepatitis	GLL0015
Qiuhaitang	Begoniaceae	*Begonia modestiflora* Kurz.	Herb	Root	Nephritis	GLL085
Sankezhen	Berberidaceae	*Berberis deinacantha* Schneid.	Shrub	Root	Heat-clearing and detoxifying, antiphlogosis, gastroenteritis, relieving cough	GLL0212
Sankezhenhuanglian	Berberidaceae	*Berberis wuliangshanens*is C.Y. Wu	Shrub	Root	Toothache	GLL0213
Chachacao	Compositae	*Bidens pilosa* Linn.	Herb	Whole plant	Heat-clearing and detoxifying, common cold, appendicitis, gastroenteritis, heat-clearing and detoxifying, laryngopharyngitis, diabetes mellitus	GLL0012
Xiaobaiji	Orchidaceae	*Bletilla formosana* (Hayata) Schltr.	Herb	Root	Relieving cough, tuberculosis	GLL0071
Baiji	Orchidaceae	*Bletilla striata* (Thunb. ex A. Murray) Rchb. f.	Herb	Stem	Pneumonia, tuberculosis, haemostasis	GLL0072
Bingpianye	Compositae	*Blumea balsamifera* (L.) DC.	Herb	Juice	Rheumatism	GLL00120
Baihucao	Rutaceae	*Boenninghausenia albiflora* (Hook.) Rchb. ex Meisn.	Herb	Whole plant, root	Antiphl titis, *exorcise evil spirits*	GLL0134
Mumian	Malvaceae	*Bombax ceiba* L.	Tree	Bark	Bone-setting, traumatic injury	GLL0163
Dabusi	Crassulaceae	*Bryophyllum pinnatum* (Lam.) Oken	Herb	Leaf, whole plant	Bone-setting, Traumatic injury	GLL070
Huanghua	Buddlejaceae	*Buddleja officinalis* Maxim.	Shrub	Root	Replenishing qi	GLL117
Chaihu	Umbelliferae	*Bupleurum hamiltonii* Balakr.	Herb	Whole plant, leaf	Common cold	GLL0111
Baichaihu	Umbelliferae	*Bupleurum marginatum* Wall.ex DC.	Herb	Whole plant	Common cold	GLL0112
Wannianqing	Buxaceae	*Buxus bodinieri* Lévl.	Shrub	Whole plant	Gastroenteritis, traumatic injury, antiphlogosis	GLL0341
Sumu	Fabaceae	*Caesalpinia sappan* Linn.	Tree	Stem	Gynecologic diseases	GLL111
Hehuanhua	Mimosaceae	*Calliandra haematocephala* Hassk.	Shrub	Flower, leaf, bark	Tranquilizing effect	GLL0332
Dawanwanhua	Convolvulaceae	*Calystegia hederacea* Wall. ex Roxb.	Herb	Whole plant	Antiphlogosis	GLL104
Chaye		*Camellia sinensis* (L.) O. Ktze.	Tree	Leaf	Antiphlogosis	GLL088
Aiqi	Liliaceae	*Campylandra wattii* C. B. Clarke	Herb	Whole plant	Gastroenteritis	GLL0056
Douling	Liliaceae	*Cardiocrinum giganteum* (Wall.) Makino	Herbaceous liane	Stem	Relieving cough, trachitis, pneumonia, emphysema	GLL0051
Xiaohonggaoliang	Cyperaceae	*Carex alta* Boott	Herb	Root	Gynecologic diseases	GLL0461
Yegaolianggen	Cyperaceae	*Carex baccans* Nees	Herb	Root	Haemostasis	GLL0462
Goujiaoji	Vitaceae	*Cayratia trifolia* (Linn.) Domin	Woody climber	Whole plant	Digestive, common cold, Heat-clearing and detoxifying	GLL0241
Jiguanhua	Amaranthaceae	*Celosia cristata* Linn.	Herb	Whole plant, flower	Heat-clearing and detoxifying, gynecologic diseases	GLL0263
Yingtaopi	Rosaceae	*Cerasus pseudocerasus* (Lindl.) G. Don	Tree	Bark	Heat-clearing and detoxifying	GLL00212
Mugua	Rosaceae	*Chaenomeles sinensis* (Thouin) Koehne	Shrub	Fruit	Rheumatism, traumatic injury	GLL0022
Suanmugua	Rosaceae	*Chaenomeles speciosa* (Sweet) Nakai	Shrub	Fruit	Rheumatism	GLL0023
Yinfencao	Sinopteridaceae	*Cheilanthes albomarginata* C.B. Clarke	Herb	Whole plant	Gynecologic diseases	GLL113
Sikuaiwa	Chloranthaceae	*Chloranthus holostegius* (Hand.-Mazz.) Pei et Shan	Herb	Whole plant, root	Common cold Laryngopharyngitis	GLL069
Tengzhong	Apocynaceae	Chonemorpha valvata	Woody climber	Stem, leaf	Rheumatism	GLL0154
Feijicao	Compositae	*Chromolaena odorata* (Linn.) R. M. King et H. Rob.	Herb	Whole plant	Gastroenteritis, heat-clearing and detoxifying	GLL00124
Santiaojin	Lauraceae	*Cinnamomum bejolghota* (Buch.-Ham.) Sweet	Tree	Bark	Ventilation, bone-setting	GLL0273
Zhangmuzi	Lauraceae	*Cinnamomum camphora* (L.) J.Presl	Tree	Fruit	Common cold, heatstroke, ventilation	GLL0275
Rougui	Lauraceae	*Cinnamomum cassia* Nees ex Blume	Tree	Bark	Ventilation, dispel coldness	GLL0272
Xiangzhang	Lauraceae	*Cinnamomum glanduliferum* (Wall.) Nees	Tree	Stem, fruit, root	Ventilation, refreshing, gastroenteritis	GLL0274
Yaluoqing	Meliaceae	*Cipadessa baccifera* (Roth.) Miq.	Tree	Leaf	Diabetes mellitus, gastroenteritis, catharsis	GLL0231
Jicigen	Compositae	*Cirsium griseum* H. Lév.	Herb	Root, leaf, whole plant	Gynecologic diseases, haemostasis, bone-setting	GLL00125
Foshougan	Rutaceae	*Citrus medica* var. *sarcodactylis* (Noot.) Swingle	Shrub	Fruit	Ventilation	GLL0131
Chenpi	Rutaceae	*Citrus reticulata* Blanco	Tree	Peel	Antiphlogosis	GLL0132
Xiaomutong	Ranunculaceae	*Clematis armandii* Franch.	Woody climber	Root	Diuretic, cystitis	GLL0125
Weilingxian	Ranunculaceae	*Clematis chinensis* Osbeck	Woody climber	Whole plant	Heat-clearing and detoxifying	GLL0124
Santiagoanyin	Verbenaceae	*Clerodendrum serratum* var. *amplexifolium* Moldenke	Shrub	Bark, leaf	Bone-setting, traumatic injury	GLL0361
Huanglian	Ranunculaceae	*Coptis chinensis* Franch.	Herb	Whole plant	Heat-clearing and detoxifying, hepatitis	GLL0121
Jijiaohuanglian	Ranunculaceae	*Coptis teeta* Wall.	Herb	Whole plant, root	Heat-clearing and detoxifying, traumatic injury	GLL0122
Shanzha	Rosaceae	*Crataegus pinnatifida* Bunge	Tree	Fruit	Digestive, hyperlipidemia	GLL0027
Naijiangcao	Compositae	*Crepis phoenix* Dunn	Herb	Whole plant	Common cold	GLL0013
Honghua	Iridaceae	*Crocus sativus* Linn.	Herb	Stamen	Traumatic injury, gynecologic diseases	GLL110
Gouxiangling	Fabaceae	*Crotalaria albida* Heyne ex Roth	Herbaceous liane	Whole plant, root	Gynecologic diseases	GLL0039
Huangguaye	Cucurbitaceae	*Cucumis sativus* Linn.	Herbaceous liane	Leaf	Antialcoholism	GLL0142
Baishu	Cupressaceae	*Cupressus funebris* Endl.	Shrub	Leaf	Heat-clearing and detoxifying, tranquilizing effect, antiphlogosis rheumatism, *exorcise evil spirits*	GLL052
Houzibeijian	Hypoxidaceae	*Curculigo capitulata* (Lour.) Kuntze	Herb	Root	Hyperosteogeny, tonifying kidney	GLL101
Huangjiang	Zingiberaceae	*Curcuma longa* Linn.	Herb	Root, stem	Hepatitis	GLL068
Wugenteng	Cuscutaceae	*Cuscuta chinensis* Lam.	Herbaceous liane	Whole plant	Ventilation, tonifying kidney	GLL099
Lushuicao	Commelinaceae	*Cyanotis vaga* (Lour.) Roem. et Schult.	Herb	Whole plant	Rheumatism	GLL0411
Xiaohonghao	Compositae	*Cyathocline purpurea* (Ham.O.Ktze) O. Kuntze.	Herb	Whole plant	Haemostasis	GLL00121
Niuxi	Amaranthaceae	*Cyathula officinalis* Kuan	Herb	Root	Lumbar muscle strain	GLL0261
Tieteng	Menispermacea	*Cyclea wattii* Diels	Woody climber	Stem	Gynecologic diseases	GLL0191
Hutoulan	Orchidaceae	*Cymbidium hookerianum* Rchb. f.	Herb	Root, stem	Traumatic injury	GLL0073
Tuoyaoyao	Asclepiadaceae	*Cynanchum otophyllum* Schneid.	Herbaceous liane	Root, stem	Lumbar muscle strain, tonifying kidney	GLL0171
Geshanxiao	Asclepiadaceae	*Cynanchum wilfordii* (Maxim.) Hemsl.	Herbaceous liane	Root	Digestive	GLL0172
Huoliangchongye	Boraginaceae	*Cynoglossum amabile* Stapf & J.R. Drumm.	Herb	Leaf	Snake venom	GLL114
Wandouxi	Fumariaceae	*Dactylicapnos scandens* (D. Don) Hutch.	Herbaceous liane	Root, whole plant	Gastroenteritis, antiphlogosis, haemostasis, digestive, hypertension, traumatic injury	GLL115
Huangcao	Orchidaceae	*Dendrobium moniliforme* (L.)Sw.	Herb	Whole plant	Improve immunity	GLL0074
Diaolanhua	Orchidaceae	*Dendrobium* nobile Lindl.	Herb	Whole plant	Bone-setting	GLL0078
Shushen	Araliaceae	*Dendropanax dentiger* (Harms) Merr.	Shrub	Whole plant	Traumatic injury	GLL0104
Yeluodisong	Fabaceae	*Desmodium griffithianum* Benth.	Herb	Whole plant	Snake venom	GLL0036
Banjiuwo	Fabaceae	*Desmodium triflorum* (Linn.) DC.	Woody climber	Whole plant	Haemostasis	GLL0035
Yuxingcao	Compositae	*Dichrocephala benthamii* C. B. Clarke	Herb	Whole plant, leaf	Headache, gastroenteritis, digestive, unknown swollen	GLL00118
Shanyangtou	Dioscoreaceae	*Dioscorea cirrhosa* Lour.	Herbaceous liane	Root	Gastroenteritis	GLL0471
Shanyao	Dioscoreaceae	*Dioscorea hemsleyi* Prain et Burkill	Herbaceous liane	Root	Tonifying kidney, replenishing Qi	GLL0472
Xuduan	Dipsacaceae	*Dipsacus asperoides* C.Y.Cheng et T.M.Ai	Herb	Root	Heat-clearing and detoxifying, bone-setting, gastroenteritis	GLL057
Wanshouzhu	Liliaceae	*Disporum cantoniense* (Lour.) Merr.	Herb	Whole plant	Replenishing qi, hysteritis, cystitis	GLL0053
Yebaihe	Liliaceae	*Diuranthera minor* (C.H. Wright) C.H. Wright ex Hemsl.	Herb	Stem	Pneumonia	GLL0052
Heliandou	Caryophyllacea	*Drymaria cordata* (L.) Willd. ex Schult.	Herb	Whole plant	Antiphlogosis, gastroenteritis	GLL118
Duyingguo	Elaeocarpaceae	*Elaeocarpus decipiens* Hemsl.	Tree	Fruit	Cholelithiasis, heat-clearing and detoxifying, gastroenteritis. Phlegm, antiphlogosis	GLL059
Jindaolifeisan	Compositae	*Elephantopus* scaber L.	Herb	Root	Asthma	GLL0011
Ciwujia	Araliaceae	*Eleutherococcus senticosus* (Rupr. et Maxim.) Maxim.	Shrub	Leaf, root, bark	Hypertension, traumatic injury, rheumatism, bone-setting, cerebral infarction, common cold. Hepatitis	GLL0105
Cisanjia	Araliaceae	*Eleutherococcus trifoliatus* (Linnaeus) S. Y. Hu	Tree	Stem	Rheumatism	GLL0107
Silenghao	Labiatae	*Elsholtzia blanda* Benth.	Herb	Whole plant	Common cold	GLL0046
Saobake	Labiatae	*Elsholtzia rugulosa* Hemsl.	Herb	Whole plant	Gastroenteritis, common cold	GLL0044
Suantengzi	Myrsinaceae	*Embelia laeta* (Linn.) Mez	Woody climber	Root	Gastroenteritis	GLL0321
Yinyanghuo	Berberidaceae	Epimedium brevicornu Maxim.	Herb	Whole plant, root, stem	Improve immunity, nephritis	GLL0214
Pashulong	Araceae	*Epipremnum pinnatum* (Linn.) Engl.	Herbaceous liane	Whole plant	Traumatic injury, bone-setting	GLL0083
Tongqicao	Equisetaceae	*Equisetum ramosissimum* Desf. subsp. *debile* (Roxb. ex Vauch.) Hauke	Herb	Whole plant, root	Ventilation, traumatic injury, cholelithiasis, heat-clearing and detoxifying, gastroenteritis	GLL081
Pipaye	Rosaceae	*Eriobotrya japonica* (Thunb.) Lindl.	Tree	Leaf	Relieving cough, common cold	GLL0024
Duzhong	Eucommiaceae	*Eucommia ulmoides* Oliv.	Tree	Bark	Traumatic injury, bone-setting nephritis, rheumatism	GLL060
Yipinhong	Euphorbiaceae	*Euphorbia cyathophora* Murr.	Herb	Whole plant	Traumatic injury	GLL0291
Candouqi	Euphorbiaceae	*Euphorbia sessiliflora* Roxb.	Herb	Whole plant	Traumatic injury, bone-setting	GLL0292
Xiaohuangsan	Rutaceae	*Evodia lepta* (Spreng.) Merr.	Tree	Leaf	Gastroenteritis Heat-clearing and detoxifying	GLL0136
Wuchuyi	Rutaceae	*Evodia rutaecarpa* (A. Juss.) Benth.	Shrub	Root, seed, whole plant	Antiphlogosis, gastroenteritis	GLL0135
Heshouwu	Polygonaceae	*Fallopia multiflora* (Thunb.) Haraldson	Herbaceous liane	Root	Digestive, enriching blood, gastroenteritis	GLL0094
Dibanteng	Moraceae	*Ficus tikoua* Bur.	Woody climber	Stem, root, whole plant, leaf	Common cold, antiphlogosis, ventilation, nephritis, gastroenteritis	GLL037
Jiayanpi	Fabaceae	*Flemingia macrophylla* (Willd.) Merr.	Shrub	Root	Gastroenteritis	GLL0034
Lalateng	Rubiaceae	*Galium aparine* Linn.	Herbaceous liane	Whole plant	Bone-setting	GLL0066
Xiaohongshen	Rubiaceae	*Galium elegans* Wall. ex Roxb. var. elegans	Herbaceous liane	Root	Bone-setting Gynecologic diseases	GLL0067
Lingzhi	Polyporaceae	*Ganoderma lucidum* (Curtis) P. Karst.	Herb	Whole plant	Improve immunity, hypertension, diabetes mellitus, ventilation, inducing diuresis	GLL061
Zhizi	Rubiaceae	*Gardenia jasminoides* Ellis	Shrub	Root	Headache	GLL0069
Tianma	Orchidaceae	*Gastrodia elata* Bl.	Herb	Root, stem	Cerebral haemorrhage	GLL0076
Ditanxiang	Ericaceae	*Gaultheria fragrantissima* Wall.	Shrub	Root, leaf	Gastroenteritis, heat-clearing and detoxifying, allergy, dermatosis, eczema	GLL0181
Gounaohua	Loganiaceae	*Gelsemium elegans* (Gardn. et Champ.) Benth.	Woody climber	Root	Heat-clearing and detoxifying	GLL077
Qinjiao	Gentianaceae	*Gentiana macrophylla* Pall.	Herb	Whole plant	Rheumatism	GLL0301
Longdancao	Gentianaceae	*Gentiana rigescens* Franch. ex Hemsl.	Herb	Whole plant, Root	Heat-clearing and detoxifying, antiphlogosis, hepatitis, gastroenteritis Gynecologic diseases, cholagogic	GLL0302
Baitouweng	Compositae	*Gerbera piloselloides* (Linn. ) Cass.	Herb	Whole plant, root	Heat-clearing and detoxifying, antiphlogosis, cervicitis	GLL00122
Shifengdan	Orchidaceae	*Goodyera psocera* HK.	Herb	Whole plant	Rheumatism, digestive	GLL0077
Yidaocao	Compositae	*Gynura divaricata* (Linn.) DC.	Herb	Leaf	Diabetes mellitus	GLL0017
Shuiganlan	Rubiaceae	*Hedyotis diffusa* Willd.	Woody climber	Whole plant, root	Heat-clearing and detoxifying, antiphlogosis, improve immunity, gastroenteritis, digestive	GLL0062
Jiegudan	Rubiaceae	*Hedyotis hedyotidea* (DC.) Merr.	Herb	Leaf	Bone-setting, traumatic injury	GLL0061
Yeshanghua	Cornaceae	*Helwingia himalaica* Hook. f. et Thoms. ex C. B. Clarke	Shrub	Leaf, whole plant	Bone-setting, traumatic injury	GLL0382
Shanbaizhi	Umbelliferae	*Heracleum barmanicum* Kurz	Herb	Root	Hypertension	GLL0113
Baizhiye	Umbelliferae	*Heracleum scabridum* Franch.	Herb	Leaf	Haemostasis	GLL0118
Guiqingcao	Gramineae	*Heteropogon contortus* (L.) P. Beauv. ex Roem. & Schult.	Herb	Whole plant	Diabetes mellitus	GLL0223
Fusanghua	Malvaceae	*Hibiscus rosa-sinensis* Linn.	Shrub	Flower	Gynecologic diseases	GLL0164
Daheifuzi	Araceae	*Homalomena occulta* (Lour.) Schott	Herb	Root, stem	Digestive, rheumatism	GLL0084
Yuxingcao	Saururaceae	*Houttuynia cordata* Thunb.	Herb	Whole plant, leaf	Gynecologic diseases, traumatic injury, expedites afterbirth, gastroenteritis, Laryngopharyngitis	GLL086
Xiaoqingteng	Hernandiaceae	*Illigera nervos* Merr.	Woody climber	Stem	Snake venom	GLL074
Huangpicao	Gramineae	*Imperata cylindrica* (L.) Raeusch.	Herb	Root	Haemostasis, replenishing qi	GLL0222
Jiagushigun	Compositae	*Inula cappa* (Buch.-Ham. ex D. Don) DC.	Herb	Root	Common cold	GLL00127
Yitong	Flacourtiaceae	*Itoa orientalis* Hemsl.	Tree	Root	Heat-clearing and detoxifying, snake venom	GLL058
Baitucao	Compositae	*Ixeris polycephala* Cass.	Herb	Whole plant	Antiphlogosis	GLL0018
yingchunhua	Oleaceae	*Jasminum nudiflorum* Lindl.	Shrub	Leaf	Heat-clearing and detoxifying	GLL080
tongxuexiang	Schisandraceae	*Kadsura heteroclita* (Roxb.) Craib	Woody climber	Root, stem	Lumbar muscle strain, rheumatism	GLL0109
Ziwei	Lythraceae	*Lagerstroemia indica* Linn.	Tree	Bark	Dermatosis, urticaria	GLL083
Choulilngdan	Compositae	*Laggera crispata* (Vahl) Hepper & J.R.I.Wood	Herb	Leaf, whole plant	Heat-clearing and detoxifying, haemostasis, snake venom, gastroenteritis, laryngopharyngitis	GLL0019
Yema	Labiatae	*Leonurus japonicus* Houtt.	Herb	Whole plant	Gynecologic diseases	GLL0048
Gezaocao	Fabaceae	*Lespedeza cuneata* (Dum. Cours.) G. Don	Shrub	Whole plant	Thrush	GLL0031
Mifengcao	Labiatae	*Leucas ciliata* Benth.	Herb	Whole plant	Rheumatism, stroke, heat-clearing and detoxifying	GLL0045
Guichuixiao	Caprifoliaceae	*Leycesteria formosa* Wall.	Shrub	Whole plant	Ventilation	GLL0251
Chuanxiong	Umbelliferae	*Ligusticum sinense* Oliv.	Herb	Root	Gynecologic diseases, traumatic injury, rheumatism	GLL0114
Lapi	Lauraceae	*Lindera tonkinensis* Lecomte var. *tonkinensis*	Tree	Bark	Ventilation	GLL0271
Jinqiancao	Campanulaceae	*Lobelia angulata* Forst.	Herbaceous liane	Whole plant	Nephritis	GLL119
Dajiangjun	Campanulaceae	*Lobelia clavata* E. Wimm.	Herb	Root	Heat-clearing and detoxifying	GLL035
Jinyinhua	Caprifoliaceae	*Lonicera maackii* (Rupr.) Maxim.	Herbaceous liane	Flower	Heat-clearing and detoxifying	GLL0252
Dingxiang	Rubiaceae	*Luculia pinceana* Hook. var. pinceana	Shrub	Bark	Rheumatism	GLL00610
Jiaogua	Cucurbitaceae	*Luffa acutangula* (Linn.) Roxb.	Herbaceous liane	Whole plant	Snake venom	GLL0144
Gouqi	Solanaceae	*Lycium chinense* Mill.	Shrub	Fruit	Gynecologic diseases, antiphlogosis, cystitis, diuretic	GLL0201
Shenjincao	Lycopodiaceae	*Lycopodium japonicum* Thunb. ex Murray	Herb	Whole plant	Bone-setting, Lumbar Muscle strain, rheumatism	GLL094
Guoluhuang	Primulaceae	*Lysimachia christiniae* Hance	Herb	Whole plant	Cholecystitis, snake venom	GLL054
Aishen	Gesneriaceae	*Lysionotus pauciflorus* var. *pauciflorus* Maxim.	Herb	Whole plant	Bone-setting	GLL073
Dashuhuanglian	Berberidaceae	*Mahonia duclouxiana* Gagnep.	Shrub	Whole plant, root, stem	Heat-clearing and detoxifying, antiphlogosis, relieving cough	GLL0211
Dabaigai	Asclepiadaceae	*Marsdenia griffithii* Hook. f.	Woody climber	Root	Heat-clearing and detoxifying, gastroenteritis, diabetes mellitus	GLL0174
Tongguangsan	Asclepiadaceae	*Marsdenia tenacissima* (Roxb.) Moon	Woody climber	Root	Digestive, antiphlogosis, rheumatism, laryngopharyngitis	GLL0175
Xiaohongteng	Urticaceae	*Memorialis hirta* (Bl.)Wedd.	Herbaceous liane	Root	Heat-clearing and detoxifying, traumatic injury	GLL0311
Haixiucao	Mimosaceae	*Mimosa pudica* Linn.	Herb	Whole plant	Rheumatism	GLL0331
Fenguo	Nyctaginaceae	*Mirabilis jalapa* Linn.	Herb	Root, whole plant	Heat-clearing and detoxifying, antiphlogosis, toothache, snake venom, diabetes mellitus, mumps	GLL116
Sangpi	Moraceae	*Morus alba* L.	Shrub	Bark, fruit, root, leaf, juice	Relieving cough, tonifying kidney, jaundice hepatitis, hyperlipidemia, laryngopharyngitis, common cold, heat-clearing and detoxifying, cholagogic, hypertension, diabetes mellitus, tonifying kidney, rheumatism	GLL120
Daxueteng	Fabaceae	*Mucuna macrobotrys* Hance	Herb	Root, stem	Bone-setting, pneumonia, relieving cough	GLL0038
Aituotuo	Meliaceae	*Munronia pinnata* (Wall.) W. Theobald	Shrub	Root	Traumatic injury	GLL0233
Yangmei	Myricaceae	*Myrica rubra* (Lour.) Siebold et Zucc.	Tree	Bark	Gastroenteritis, analgesic	GLL106
Jingjie	Labiatae	*Nepeta cataria* Linn.	Herb	Whole plant	Haemostasis, common cold	GLL0042
Shuiqincai	Umbelliferae	*Oenanthe javanica* (Bl.) DC.	Herb	Whole plant	Hypertension	GLL0116
Babaozhenxindan	Liliaceae	*Ophiopogon dracaenoides* (Baker)HK.f.	Herb	Whole plant	Heart disease	GLL0057
Xianrenzhang	Cactaceae	*Opuntia dillenii* (Ker Gawl.) Haw.	Herb	Stem	Antiphlogosis, unknown swollen, *exorcise evil spirits*	GLL102
Haichuang	Bignoniaceae	*Oroxylum indicum* (Linn.) Kurz	Tree	Fruit	Hepatitis	GLL0421
Chaotianguan	Melastomataceae	*Osbeckia crinita* Benth. ex C. B. Clarke	Shrub	Whole plant	Hepatitis	GLL107
Laowasuanyingcai	Oxalidaceae	*Oxalis corniculata* Linn.	Herb	Whole plant	Rheumatism, gynecologic diseases, nephritis, gastroenteritis, Migraine, heat-clearing and detoxifying, traumatic injury, haemostasis	GLL0452
Honghuadiding	Oxalidaceae	*Oxalis corymbosa* DC.	Herb	Whole plant	Traumatic injury, heat-clearing and detoxifying	GLL0451
Jishiteng	Rubiaceae	*Paederia foetida* Linn.	Herbaceous liane	Stem, leaf	Antiphlogosis	GLL0065
Mudanhua	Paeoniaceae	*Paeonia suffruticosa* Andr.	Shrub	Root	Heart disease, neurasthenia	GLL091
Sanqi	Araliaceae	*Panax pseudo-ginseng* Wall.	Herb	Root	Hypertension, traumatic injury, lumbar muscle strain	GLL0102
Yesanqi	Araliaceae	*Panax zingiberensis* C. Y. Wu et K. M. Feng	Herb	Root	Traumatic injury, bone-setting	GLL0103
Yingsu	Papaveraceae	*Papaver somniferum* Linn.	Herb	Nutshell, fruit	Gastroenteritis, antiphlogosis	GLL109
Chonglou	Liliaceae	*Paris polyphylla* Smith	Herb	Root	Traumatic injury haemostasis, unknown swollen, antiphlogosis, gastroenteritis	GLL0055
Sanxuedan	Piperaceae	*Peperomia blanda* (Jacq.) Kunth	Herb	Whole plant	Traumatic injury	GLL0281
Suzi	Labiatae	*Perilla frutescens* var. *purpurascens* (Hayata) H.W. Li	Herb	Whole plant	Relieving cough	GLL0049
Fengteng	Asclepiadaceae	*Periploca calophylla* (Woght) Falc.	Woody climber	Leaf, whole plant, Stem	Heat-clearing and detoxifying, antiphlogosis rheumatism	GLL0173
Ganlanguo	Euphorbiaceae	*Phyllanthus emblica* Linn.	Tree	Bark, fruit	Gastroenteritis, hyperlipidemia	GLL0293
Shanglu	Phytolaccaceae	*Phytolacca americana* Linn.	Herb	Whole plant	Heat-clearing and detoxifying, diuretic	GLL090
Dafangfeng	Umbelliferae	*Pimpinella candolleana* Wight et Arn.	Herb	Whole plant	Digestive, antiparastics	GLL0115
Banxia	Araceae	*Pinellia ternata* (Thunb.) Makino	Herb	Root	Heat-clearing and detoxifying	GLL0081
Simaosong	Pinaceae	*Pinus kesiya Royle* ex Gordon	Tree	Branch, leaf, root	Catharsis, traumatic injury, *exorcise evil spirits*	GLL096
Yezilan	Piperaceae	*Piper boehmeriaefoliu*m (Miq.) C. DC.	Shrub	Fruit, whole plant, root, stem	Digestive, common cold, gastroenteritist, raumatic injury, bone-setting, rheumatism	GLL0284
Waiyezilan	Piperaceae	*Piper boehmeriaefolium* var. *tonkinense* C. DC.	Tree	Whole plant	Rheumatism	GLL0283
Yehujiao	Piperaceae	*Piper nigrum* Linn.	Tree	Root, bark, fruit	Antiphlogosis	GLL0282
Laihamacao	Plantaginaceae	*Plantago minuta* Pall.	Herb	Whole plant	Heat-clearing and detoxifying, common cold, antiphlogosis, cystitis, Prostatitis	GLL056
Baihuadan	Plumbaginaceae	*Plumbago zeylanica* Linn.	Herb	Root	Traumatic injury	GLL050
Jidanhua	Apocynaceae	*Plumeria rubra* Linn.	Shrub	Leaf	Lumbar muscle strain, traumatic injury	GLL0151
Jiduzishu	Polygalaceae	*Polygala arillata* Buch.-Ham. ex D. Don	Shrub	Root	Gynecologic diseases, digestive	GLL0482
Hongbeilan	Polygalaceae	*Polygala latouchei* Franch.	Tree	Whole plant	Heat-clearing and detoxifying	GLL0481
Suanjiangcao	Polygonaceae	*Polygonum capitatum* Buch.-Ham. ex D. Don	Herb	Whole plant	Bone-setting, traumatic injury checking diarrhoea, haemostasis	GLL0091
Huzhang	Polygonaceae	*Reynoutria japonica* Houtt.	Herb	Whole plant	Traumatic injury	GLL0097
Gongyaolao	Polygonaceae	*Polygonum paleaceum* Wall. ex Hook. f.	Herb	Root	Lumbar muscle strain, nephritis, traumatic injury	GLL0092
Sanxuelan	Polygonaceae	*Polygonum runcinatum* Buch.-Ham. ex D. Don var. sinense Hemsl.	Herb	Whole plant	Traumatic injury	GLL0093
Machixian	Portulacaceae	*Portulaca oleracea* Linn.	Herb	Whole plant	Traumatic injury, hypertension	GLL076
Dibinlang	Rosaceae	*Potentilla fulgens* Wall. ex Hook.	Herb	Fruit	Digestive	GLL0029
Fanbaiye	Rosaceae	*Potentilla lineata* Trevir.	Herb	Whole plant, root	Heat-clearing and detoxifying, gastroenteritis, digestive, dysentery	GLL00210
Xiakucao	Labiatae	*Prunella vulgaris* Linn.	Herb	Whole plant	Heat-clearing and detoxifying, antiphlogosis, hepatitis, hypertension	GLL0043
Fanshiliu	Myrtaceae	*Psidium guajava* Linn.	Tree	Leaf	Gastroenteritis	GLL097
Fengweicao	Pteridaceae	*Pteris multifida* Poir.	Herb	Whole plant	Dog bite	GLL062
Gegen	Fabaceae	*Pueraria montana* var. *lobata* (Willd.) Maesen et S. M. Almeida ex Sanjappa et Predeep	Shrub	Root	Common cold, snake venom, antialcoholism	GLL00310
Shiliuhua	Punicaceae	*Punica granatum* Linn.	Tree	Flower, fruit, bark	Gynecologic diseases, cholelithiasis	GLL093
Mali	Fagaceae	*Quercus acutissima* Carruth.	Tree	Bark, root, leaf	Lumbar muscle strain, gastroenteritis	GLL072
Luobo	Cruciferae	*Raphanus sativus* Linn.	Herb	Root, stem	Common cold	GLL092
Luofumu	Apocynaceae	*Rauvolfia verticillata* (Lour.) Baill.	Shrub	Root, leaf	Hypertension	GLL0153
Guoshanlong	Araceae	*Rhaphidophora lancifolia* Schott	Herbaceous liane	Root	Bone-setting	GLL0085
Dahuang	Polygonaceae	*Rheum officinale* Baill.	Herb	Root	Catharsis, checking diarrhoea	GLL0096
Huixincao	Bryaceae	*Rhodobryum roseum* Limpr.	Herb	Whole plant	Heart disease	GLL112
Dujuanhua	Ericaceae	*Rhododendron delavayi* Franch.	Shrub	Flower	Gynecologic diseases	GLL0183
Yueji	Rosaceae	*Rosa chinensis* Jacq.	Shrub	Flower	Gynecologic diseases	GLL0026
Jinyingzi	Rosaceae	*Rosa laevigata* Michx.	Woody climber	Root, fruit	Gastroenteritis	GLL0025
Nianniancao	Rubiaceae	*Rubia cordifolia* L.	Herbaceous liane	Root	Haemostasis	GLL0068
Huangciguo	Rosaceae	*Rubus ellipticus* var. *obcordatus* (Franch.) Focke	Shrub	Root, leaf	Tonic, gastroenteritis	GLL00211
Tudahuang	Polygonaceae	*Rumex dentatus* Linn.	Herb	Whole plant	Gastroenteritis	GLL0095
Qingfengteng	Sabiaceae	*Sabia yunnanensis* Franch.	Woody climber	Stem, leaf	Heat-clearing and detoxifying	GLL084
Liushupi	Salicaceae	*Salix matsudana* Koidz.	Tree	Bark	Stroke	GLL105
Xuepencao	Caprifoliaceae	*Sambucus javanica* Reinw. ex Blume	Shrub	Whole plant, leaf	Bone-setting, traumatic injury, rheumatism	GLL0253
Yeshanhua	Buxaceae	*Sarcococca ruscifolia* Stapf	Shrub	Root	Bone-setting	GLL0342
Qiyelian	Araliaceae	*Schefflera arboricola* Hayata	Shrub	Leaf	Traumatic injury	GLL0101
Xiaohongteng	Schisandraceae	*Schisandra henryi* C.B.Clarke	Woody climber	Root, fruit	Heat-clearing and detoxifying, heat-clearing and detoxifying, rheumatism	GLL0108
Zuandifeng	Saxifragaceae	*Schizophragma integrifolium* Oliv.	Woody climber	Root	Heat-clearing and detoxifying, traumatic injury, rheumatism	GLL066
Huojieyao	Compositae	*Scorzonera ikonnikovii* Lipsch. et Krasch. ex Lipsch.	Herb	Whole plant	Heat-clearing and detoxifying	GLL00117
Yizhijian	Labiatae	*Scutellaria discolor* Colebr.	Herb	Whole plant, Root	Antiphlogosis, relieving cough	GLL0047
Jiuliguang	Compositae	*Senecio scandens* Buch.-Ham. ex D. Don	Herb	Root	Heat-clearing and detoxifying, antiphlogosis	GLL00112
Xiaohuaishu	Fabaceae	*Senna occidentalis* (L.) Link	Tree	Flower	Haemostasis	GLL00311
Yehuasheng	Fabaceae	*Senna tora* (L.) Roxb.	Shrub	Whole plant	Snake venom	GLL0032
Baduyao	Malvaceae	*Sida acuta* Burm. f.	Shrub	Root	Traumatic injury, unknown swollen	GLL0161
Tufuling	Smilacaceae	*Smilax glabra* Roxb.	Shrub	Root	Gynecologic diseases	GLL049
Xiwanshu	Solanaceae	*Solanum donianum* Walp.	Tree	Root	Common cold	GLL0203
Kuliangcai	Solanaceae	*Solanum nigrum* Linn.	Herb	Whole plant	Heat-clearing and detoxifying, traumatic injury, haemostasis	GLL0202
Laoshuhuanggua	Cucurbitaceae	*Solena amplexicaulis* (Lam.) Gandhi	Herbaceous liane	Root	Diabetes mellitus, antiphlogosis, tonsillitis	GLL0143
Huaishu	Fabaceae	*Sophora japonica* Linn.	Shrub	Flower	Haemostasis	GLL0033
Huibaocao	Caryophyllacea	*Stellaria vestita* Kurz var. vestita	Herb	Whole plant	Heat-clearing and detoxifying, traumatic injury, haemostasis, bone-setting	GLL039
Jiuguniu	Stemonaceae	*Stemona tuberosa* Lour.	Herb	Root	Phlegm, replenishing qi	GLL051
Shanwugui	Menispermacea	*Stephania delavayi* Diels	Herbaceous liane	Root	Digestive, antiphlogosis, gastroenteritis, analgesic	GLL0193
Juhuaxin	Menispermacea	*Stephania tetrandra* S. Moore	Herbaceous liane	Root	Gastroenteritis	GLL0192
Banlangen	Acanthaceae	*Strobilanthes cusia* (Nees) J.B.Imlay	Herb	Root, leaf	Common cold, antiphlogosis, gastroenteritis	GLL071
Yudancao	Gentianaceae	*Swertia bimaculata* (Sieb.et Zucc.)Hook.f.et Thoms.	Herb	Whole plant	Hepatitis, cholecystitis	GLL0303
Xiaoheke	Symplocaceae	*Symplocos paniculata* (Thunb.) Miq.	Shrub	Whole plant	Common cold	GLL089
Huanghualam	Compositae	*Taraxacum mongolicum* Hand.-Mazz.	Herbaceous liane	Whole plant	Heat-clearing and detoxifying, antiphlogosis, analgesic, breast cancer	GLL00111
Sangjisheng	Loranthaceae	*Taxillus sutchuenensis* (Lecomte) Danser	Shrub	Whole plant	Tonifying kidney, rheumatism, antiphlogosis	GLL087
Hongduoshan	Taxaceae	*Taxus wallichiana* Zucc.	Tree	Bark	Antiparastics	GLL064
Zhulinbiao	Bignoniaceae	*Tecoma capensis* (Thunb.) Lindl.	Woody climber	Whole plant, stem	Lumbar muscle strain	GLL0422
Wuzhuajinlong	Vitaceae	*Tetrastigma hypoglaucum* Planch.	Woody climber	Whole plant	Traumatic injury	GLL0243
Huanglian	Ranunculaceae	*Thalictrum foliolosum* DC.	Herb	Whole plant	Gastroenteritis	GLL0123
Luoguodi	Cucurbitaceae	*Thladiantha villosula* Cogn.	Herbaceous liane	Root, stem, whole plant	Heat-clearing and detoxifying, gastroenteritis, antiphlogosis	GLL0141
Aijiao	Orchidaceae	*Thunia alba* (Lindl.) Rchb. f.	Herb	Root, stem, whole plant	Traumatic injury bone-setting	GLL0075
Jinxiandiaohulu	Menispermacea	*Tinospora sagittata* (Oliv.) Gagnep.	Herbaceous liane	Root	Heat-clearing and detoxifying, analgesic, unknown swollen, gastroenteritis	GLL0194
Xiangchun	Meliaceae	*Toona sinensis* (A. Juss.) Roem.	Tree	Root, bark	Heat-clearing and detoxifying, allergy	GLL0232
Laomianguashu	Cornaceae	*Toricellia tiliifolia* DC.	Tree	Leaf	Nephritis	GLL0381
Zongshu	Palmae	*Trachycarpus fortunei* (Hook.) H. Wendl.	Tree	Root	Traumatic injury	GLL0432
Zizhumei	Commelinaceae	*Tradescantia pallida* (Rose) D.R.Hunt	Herb	Whole plant	Antiphlogosis	GLL0412
Yiner	Tremellaceae	*Tremella fuciformis*		Whole plant	Tonic	GLL108
Citong	Araliaceae	*Trevesia palmata* (Roxb.) Vis.	Shrub	Root, bark	Bone-setting, traumatic injury	GLL0106
Leigongteng	Celastraceae	*Tripterygium wilfordii* Hook. f.	Shrub	Stem, leaf	Liver cancer	GLL100
Jinsiling	Tropaeolaceae	*Tropaeolum majus* Linn.	Herb	Whole plant	Otitis	GLL063
Gaojiaoaiqi	Liliaceae	*Tupistra aurantiaca* Wall.ex Baker	Herb	Whole plant	Bone-setting, gastroenteritis	GLL0054
Baibuhuanyuan	Araceae	*Typhonium blumei* Nicolson et Sivadasan	Herb	Whole plant	Laryngopharyngitis, snake venom, heat-clearing and detoxifying	GLL0086
Yutouqi	Araceae	*Typhonium divaricatum* (L.) Decne	Herb	Stem	Gastroenteritis	GLL0087
Jingou	Rubiaceae	*Uncaria laevigata* Wall. ex G. Don	Woody climber	Root, stem	Traumatic injury	GLL0064
Gouteng	Rubiaceae	*Uncaria rhynchophylla* (Miq.) Miq. ex Havil.	Woody climber	Root, stem	Heat-clearing and detoxifying	GLL0063
Xiqianma	Urticaceae	*Urtica angustifolia* Fisch. ex Hornem.	Herb	Whole plant	Rheumatism	GLL0312
Xiezicao	Urticaceae	*Urtica fissa* E. Pritz.	Herb	Whole plant	Rheumatism, urticaria	GLL0313
Matixiang	Valerianaceae	*Valeriana jatamansi* Jones	Herb	Whole plant	Gastroenteritis	GLL053
Xiaozongbao	Liliaceae	*Veratrum mengtzeanum* Loes. f.	Herb	Root, stem	Antiparastics	GLL0058
Mabiancao	Verbenaceae	*Verbena officinalis* Linn.	Herb	Root, whole plant	Common cold, heat-clearing and detoxifying, gastroenteritis	GLL0362
Dashufasan	Compositae	*Vernonia parishii* Hook. f.	Herb	Root	Common cold	GLL00114
Pangxiejiao	Viscaceae	*Viscum articulatum* Burm. f.	Herb	Whole plant	Antiphlogosis, cystitis	GLL065
Yantong	Scrophulariaceae	*Wightia speciosissima* (D.Don)Merr.	Tree	Bark	Bone-setting	GLL103
Yulan	Magnoliaceae	*Yulania denudata* (Desr.) D. L. Fu	Tree	Flower	Headache	GLL078
Huajiao	Rutaceae	*Zanthoxylum bungeanum* Maxim.	Shrub	Bark, fruit, root	Toothache, antiphlogosis	GLL0133
Yumixu	Gramineae	*Zea mays* Linn.	Herb	Stamen	Hypertension, diuretic	GLL0221
Shuixianhua	Amaryllidaceae	*Zephyranthes carinata* Herb.	Herb	Root	Antiphlogosis	GLL095

**Fig. 2:**
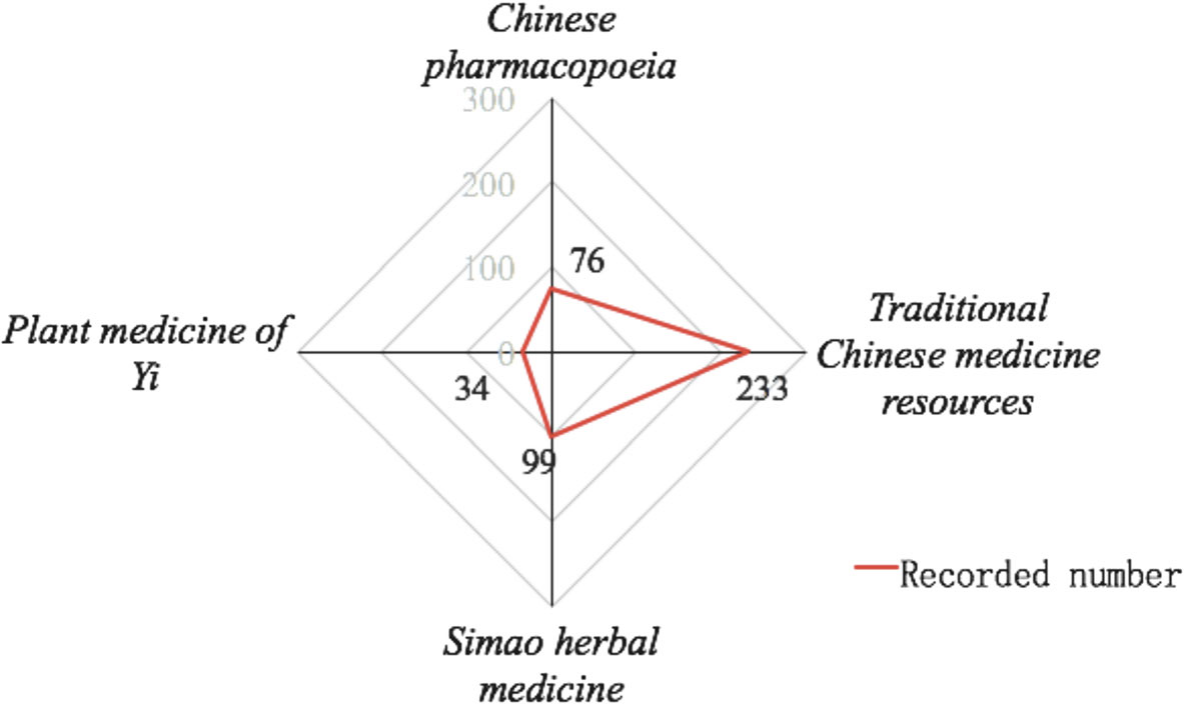
Document evidence of traditional medicinal plants in the study area

The traditional medicinal plants used in the study area possess a high ratio of being documented in the literature. Of all 302 species, 76 were recorded in the *Chinese Pharmacopoeia,* which is an authoritative masterwork in China, and 233 species were recorded in *Traditional Chinese Medicine Resources*. The local medicine journal *Plant Medicine of Yi* and *Simao Herbal Medicine* recorded 34 and 99 species, respectively (Fig. 2).

According to the analysis of the constitution of medicinal plants, the single-species family and the single-species genus had an absolute advantage in number (Tables 3 and 4), indicating that the medicinal plants in this region have high diversity in the composition of species at the family and genus level, which is similar to the survey of Shen [13].

**Table 3:** The statistics of traditional medicinal plants at the family level

Number of species within one family	Number of families	Ratio (%)	Number of species	Ratio (%)
1 species	69	58.97	69	22.85
2 to 5 species	36	30.77	107	35.43
6 to 9 species	7	5.98	56	18.54
Over 10 species	5	4.27	70	23.18

**Table 4:** The statistics of traditional medicinal plants at the genus level

Number of species within one genus	Number of genera	Ratio (%)	Number of species	Ratio (%)
1 species	212	84.13	212	70.20
2 species	34	13.49	68	22.52
3 species	2	0.79	6	1.99
4 species	4	1.59	16	5.30

**Fig. 3:**
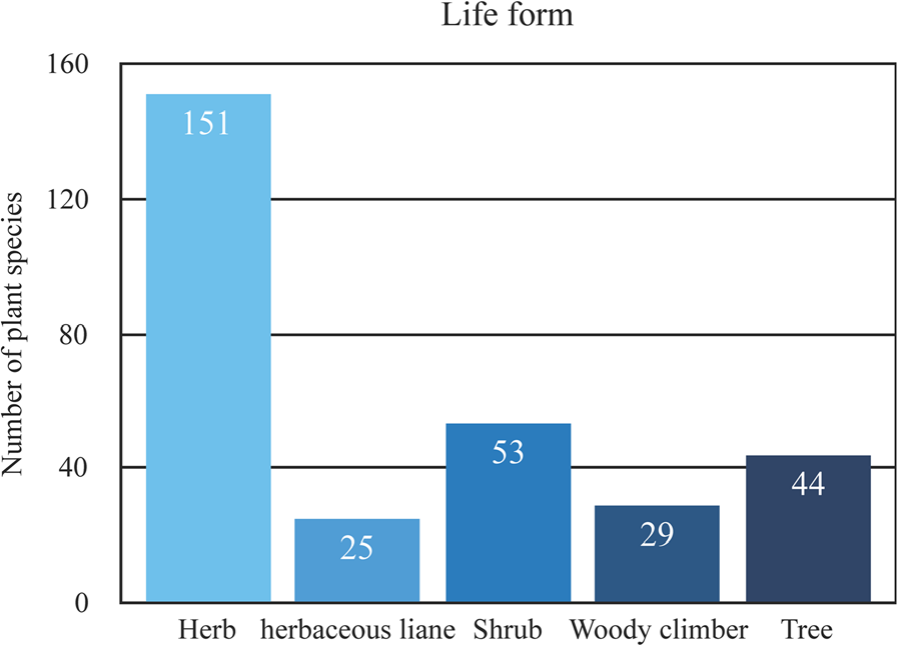
Life forms of medicinal plants in the study area

In Fig. 3, the life form analysis of traditional medicinal plants showed that herbaceous plants constituted the highest proportion, represented by 151 (50%) species, while there were 53 (17.55%) shrub species, 25 (8.28%) herbaceous lianas, 29 (9.60%) woody climbers and 44 (14.57%) tree species. This result is similar to the study of Lisu community in Nujiang, which is a minority community of China and lives in the Hengduan Mountains area as well [14, 15]. The main reason why herbs are the main medicinal plants is likely due to their diversity and convenience.

**Fig. 4:**
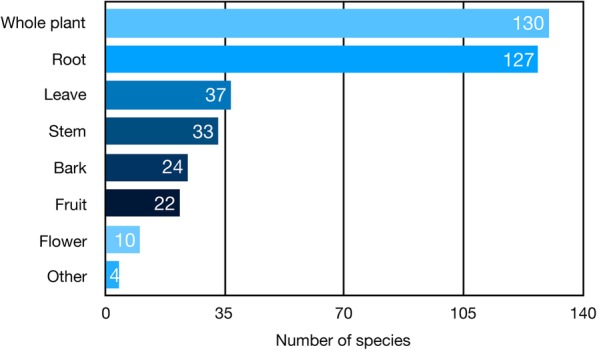
Parts of the plants used for medicinal purposes in the study area

Informants in the study area used different plant parts for the preparation of traditional drugs. Based on the data from informants, the majority of the traditional medicinal plant species were harvested as a whole plant (130), followed by the roots (127), leaves (37), stems (33), bark (24), fruits (22), flowers (10) and other parts (4) (Fig. 4). However, some studies suggest that this mode of utilization may lead to the depletion of traditional medicinal resources [16, 17].

**Fig. 5:**
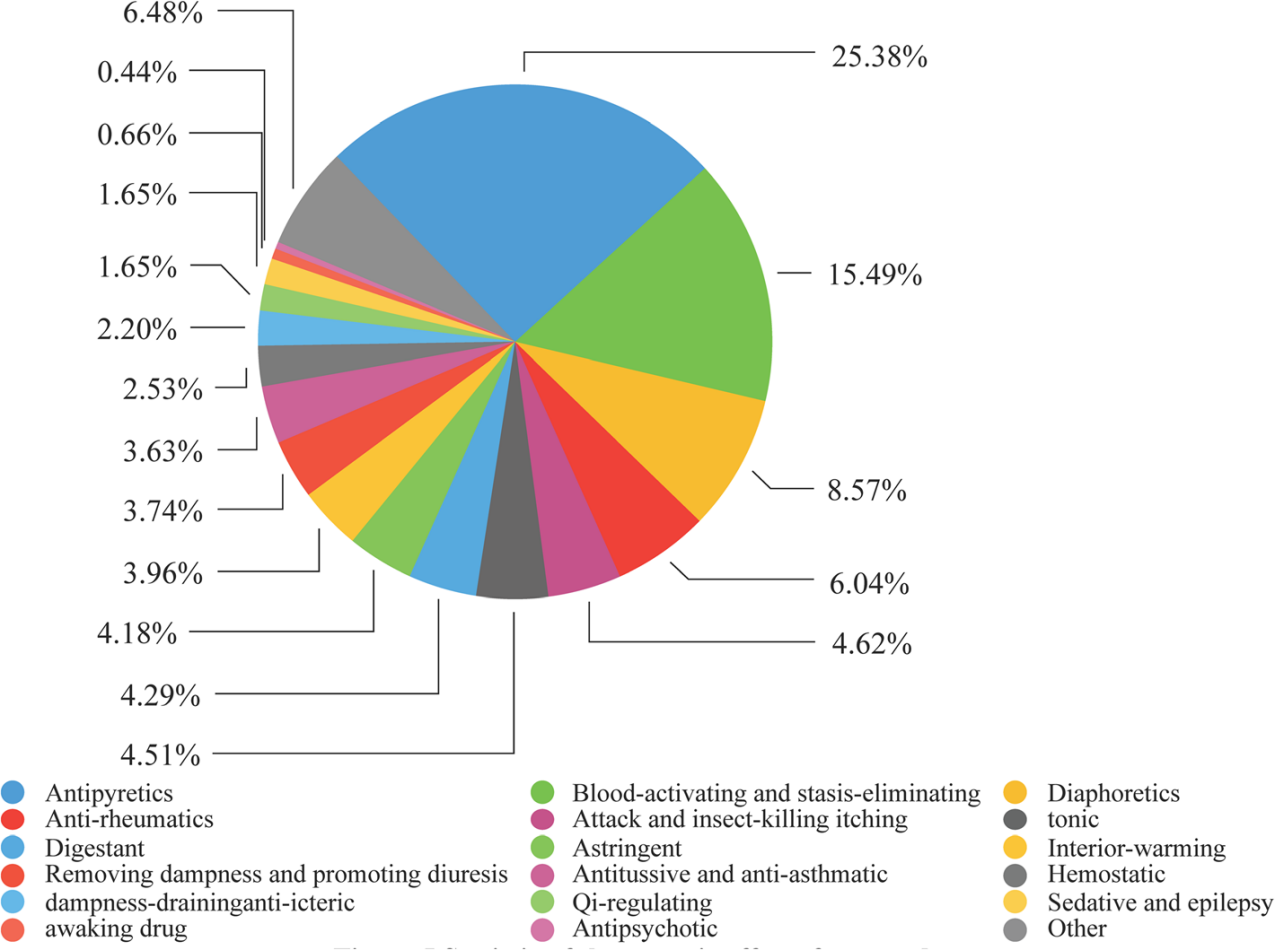
Statistic of therapeutic effects from the study area

Efficacy analysis of traditional medicinal plants was carried out based on *Chinese Medicinal Materials* [18]. The results showed that the medicinal plants were used for treating 93 human ailments in the study area. Antipyretics drugs occupy the highest proportion, followed by activating blood and eliminating stasis, diaphoretics and antirheumatics (Fig. 5). This result differed from the study of medicinal plants used by the Yi ethnic group in Chuxiong of Yunnan, showed that trauma was the most common disease. The particular geology and climate are ideal for unique Yi medicine effective in treating pyretic toxicity, rheumatism and other ailments [14].

### Endangered category assessment

**Fig. 6:**
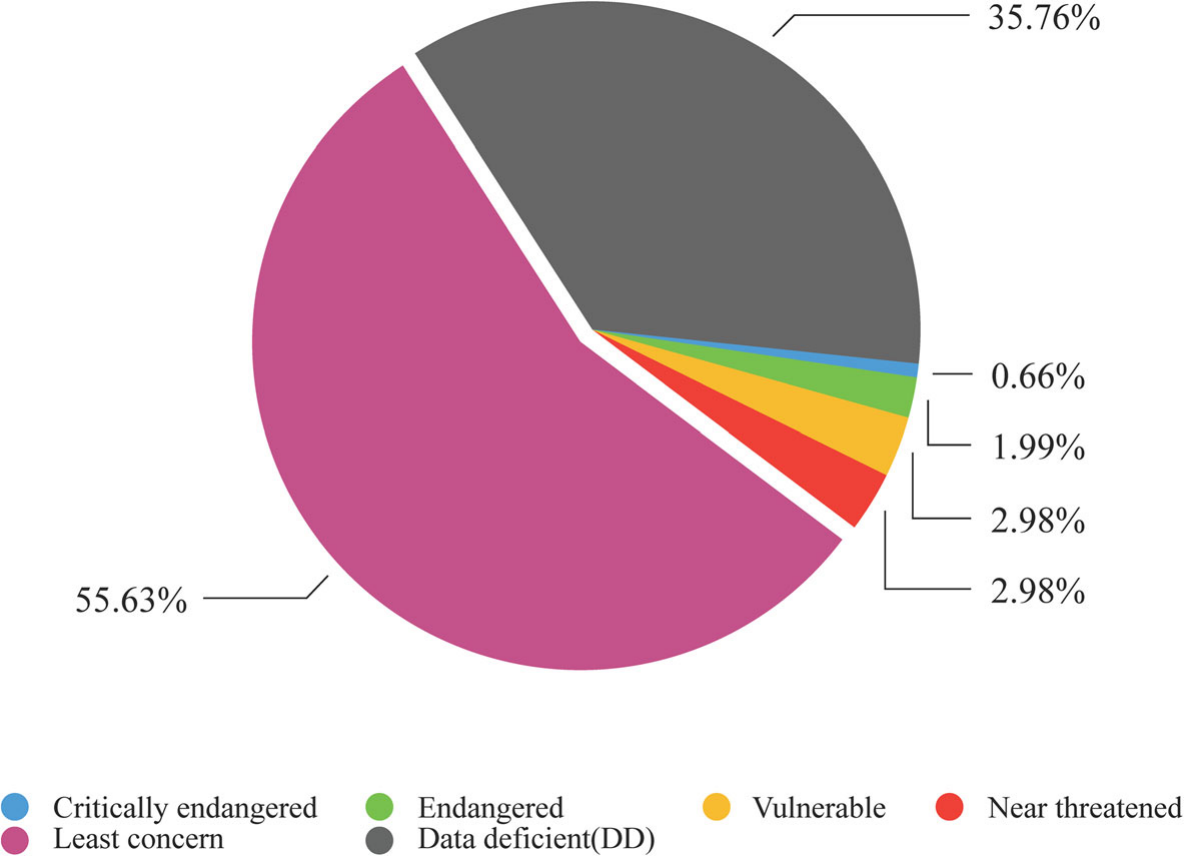
Endangerment level of traditional medicinal plants in the study area

According to the *Red List of Chinese Biodiversity (Higher Plant Volume)* [19], the level of endangerment of the traditional medicinal plants in the study area was assessed. The ratio of endangered species of traditional medicinal plants in the Jingdong Yi community area (Fig. 6) was higher than that in the Wuliang Mountains National Nature Reserve but lower than that observed nationwide [20], which does not suggest that the harvest of traditional medicinal plants by local people to treat disease is the main reason for their decrease.

### Comparison differences of medicinal plants between Yi and Han communities

The Yi and Han communities in the study area have lived in the Yi autonomous county of Jingdong in a multi-ethnic association for many years. When comparing their traditional medicinal plants, an extremely dissimilar relationship was found. The Jaccard similarity index was 0.232, which indicated a low degree of medicinal species overlap between the two communities. This result could be explained by the viewpoint that different cultural backgrounds play an essential role in the utilization of traditional medicinal plants [21]. Comparisons of different communities within the same area proved that a massive discrepancy in terms of traditional medicinal plants still exists even after being fused for a long time. Therefore, the national specificity in the utilization of medicinal plants persists in the region and modern society as well [22]. However, more ethnobotanical documentation research from Yunan Province have shown that minority’s medicinal culture is facing the increasing danger of dying out, under the great impact from Han community’s culture and way of life [4, 5, 23].

## Conclusion

This is the first ethnobotanical study conducted in the Wuliang Mountains of Jingdong, and a total of 302 species were recorded. The results show a high diversity of traditional medicinal plants, as we previously suspected. By assessment of endangered status, the traditional medicinal plants in the study area exhibit excellent conditions. This indicates that folk utilization is not the main reason for the degeneration of wild resources. The use of a large number of certain herbs as merchandize may contribute to the deteriorating situation of wild medicinal plants, such as the reduction of *Panax notoginseng* (Burkill) F.H. Chen ex C. Chow & W.G. Huang and *Paris polyphylla* var. *yunnanensis* (Franchet) Handel-Mazzetti. In contrast, some minority communities have traditional methods to protect their precious wild resources. For example, the Red-Headed Yao People in China select different parts of medicinal plants to treat diseases and selectively harvest old roots, leaving the new roots, according to different seasons and climatic conditions [24]. The Yi community in Jingdong Autonomous County also has a belief in nature, which plays a vital role in the sustainable utilization of wild resources. They have a belief of animism and believe that every tree is divine and thus deserves to be protected and respected. The people who engage in the destruction of the sacred trees have a fear of future retaliation and punishment [25].

Despite the abundance of medicinal plants in the study area, the inheritance of this valuable culture is facing a serious threat, mainly due to the rapid development of modern medicine. The ageing of herbalists without inheritors results in the rapid loss of valuable knowledge. In addition, the knowledge of traditional medicinal plants in Jingdong inherited via the oral mode and the accuracy of inheritance are difficult to determine. The most critical challenge is the lack of wild resources. According to statistics, approximately 96% of traditional medicinal plants come from the wild [26]. Especially in China, with the increasing demand for resources, tremendous pressure from overexploitation is faced by many regions. Hence, these regions should take some effective measures to protect these valuable resources and maintain their sustainable utilization in the future.

As one of the birthplaces of Yi medicine, knowledge about traditional medicinal plants is infinite, and it is a precious wealth left behind by ancestors. With regard to the application of these species, there are still many limitations that should be addressed and improved by modern science and techniques.

## Additional file

Additional file 1: Investigated sites in the study area. (DOCX 14 kb)

## Data Availability

We are willing to share the data generated and analysed during the current study.

## References

[1] Calixto JB (2005). Twenty-five years of research on medicinal plants in Latin America. a personal view. J Ethnopharmacol..

[2] Au DT, Wu J, Jiang Z (2008). Ethnobotanical study of medicinal plants used by Hakka in Guangdong, China. J Ethnopharmacol..

[3] Li DL, Xing FW (2016). Ethnobotanical study on medicinal plants used by local Hoklos people on Hainan Island, China. J Ethnopharmacol..

[4] Liu CY, Dao ZL, Yang CY, Long CL (2009). Medicinal plants used by Tibetans in Shangrila, Yunnan, China. J Ethnobiol and Ethnomed..

[5] Liu YJ, Ahmed S, Liu B, Guo ZY, Huang WJ, Wu XJ, Li SH, Zhou JJ, Lei QY, Long CL (2014). Ethnobotany of dye plants in Dong communities of China. J Ethnobiol and Ethnomed..

[6] Han YL, Zhao SY, Zhou Y (2017). The modern transition of Yi medicine inheritance patterns. Yunnan J Tradit Chin Med and Mater Medica..

[7] Wang M, Zhu JY (1998). Yi medicinal plants of Chuxiong.

[8] Xiong JR (2012). A brief history of Jingdong Yi community.

[9] Peng H (1998). The seed plants from Mt. Wuliang in the south-central.

[10] Peng CZ, Ji CB (2007). Folk proved recipe of Yi ethnomedicine in Jingdong. J Med and Pharm of Chin Minorities..

[11] Local chronicles Editorial board of Jingdong Yi autonomous county (2016). Jingdong Yearbook.

[12] Delectis Florae Reipublicae (1959). Popularis Sinicae Agendae Academiae Sinicae (DFRPS Edita). Flora Republicae Popularis Sinicae.

[13] Shen SK, Wu FQ, Zhang AL, Lin RT, Zhang XJ, Yang GS, Wang YH (2014). Germplasm resources and diversity of medicinal vascular plants in Tengchong County, Yunnan Province. Plant Sci J..

[14] Long CL, Li SM, Bo L, Shi YN, Liu BX (2009). Medicinal plants used by the Yi ethnic group: a case study in central Yunnan. J Ethnobiol and Ethnomed..

[15] Ji H, Pei SJ, Long CL. An ethnobotanical study of medicinal plants used by the Lisu people in Nujiang, northwest Yunnan, China. Econ Bot. 2004;58(Suppl)11:253–264 https://doi.org/10.1663/0013-0001(2004)58%5BS253:AESOMP% 5D2.0.CO;2

[16] FAO Inter-Departmental Working Group on Biological Diversity for Food and Agriculture (2003). Biodiversity and the ecosystem approach in agriculture, forestry and fisheries.

[17] Tjeck Olga, Souza Alain, Mickala Patrick, Lepengue Alexis, MBatchi Bertrand (2017). Bio-efficacy of medicinal plants used for the management of diabetes mellitus in Gabon. An ethnopharmacological approach. Journal of Intercultural Ethnopharmacology.

[18] Zhong GS, Wan F (2008). Chinese medicinal materials.

[19] Ministry of Environmental Protection and Chinese Academy of Sciences. Red list of Chinese biodiversity in higher plants; 2013.

[20] Gao LL, Yang HJ, Liu GZ, Wang CH, Yang GP, Cai CT (2018). Diversity of medicinal vascular plant resources in Wuliangshan National Nature Reserve, Yunnan, China. Plant Sci J..

[21] Leonti M, Nebel S, Rivera D, Heinrich M (2006). Wild gathered food plants in the european mediterranean. A comparative analysis. Econ Bot..

[22] Weckerle CS, Ineichen R, Huber FK, Yang Y (2009). Mao’s heritage: Medicinal plant knowledge among the Bai in Shaxi, China, at a crossroads between distinct local and common widespread practice. J Ethnopharmacol..

[23] Huang WJ, Li P, Liu YJ, Huang W, Ju Y, Wang JJ, Ntumwel CB, Long CL. Ethnobotanical study on medicinal plants used by Li people in Ledong, Hainan Island, China. Acta Soc Bot Pol. 2016;85:3485–99. http://ir.kib.ac.cn/handle/151853/2621

[24] Long CL, Rong L (2004). Ethnobotanical studies on medicinal plants used by the Red-headed Yao People in Jinping, Yunnan Province, China. J Ethnopharmacol..

[25] Liu JL, Zhang MH, Zhang RH (2015). Livelihoods, culture and traditional forest-related knowledge of Yi People in Yunan Province: a case study of Nanhua County. Chin Agric Univ J Soc Sci Ed..

[26] S.E.P.A (1997). National status report on biodiversity of China, status environment protection agency (SEPA).

