# Supplementary material for: Ethnomedicine study on traditional medicinal plants in the Wuliang Mountains of Jingdong, Yunnan, China
Source: J Ethnobiol Ethnomed. 2019 Aug 19;15:41. doi: 10.1186/s13002-019-0316-1 (PMC6699132; doi:10.1186/s13002-019-0316-1)
Supplement: Supplementary file 1 — Investigated sites in the study area. (DOCX 14 kb) [file 13002_2019_316_MOESM1_ESM.docx]

Supplenment 1 Investigated sites in the study area

| Town name | Village name |
| --- | --- |
| Linjie | Dajie, Kungang, |
| Xiaolongjie | Guowa, Baizhulin, Baishui |
| Jinping | Jinping |
| Taizhong | Huangcaoba |
| Jingfu | Qincaitang, Menglingjie, Shangchanghe |
| Anding | Yicang, Zhongcang, Shujie |
| Manwan | Manwan, Xia, Wenmao |
